# Toward feminist principles for epidemiology and public health

**DOI:** 10.3389/fpubh.2026.1809934

**Published:** 2026-04-16

**Authors:** Stephanie Batram-Zantvoort

**Affiliations:** School of Public Health, Department of Epidemiology and International Public Health, Bielefeld University, Bielefeld, Germany

**Keywords:** feminist epidemiology, gender, health equity, intersectionality, mixed methods, reflexivity, social determinants of health

## Abstract

**Background:**

Epidemiology has traditionally examined the distribution and determinants of health in populations. While early work focused on social determinants and collective conditions, recent decades have seen a shift toward individualized, molecular, and risk-based models emphasizing behavior, biology, and lifestyle. This shift has often obscured structural determinants and reinforced a biomedical paradigm. Feminist scholarship critiques these trends by centering lived experiences, power dynamics, and inequities in health research.

**Main body:**

This commentary explores how feminist principles can inform epidemiology, and Public Health, bridging the divide between positivist health sciences and feminist epistemologies. It outlines key feminist contributions including epistemology, theory, methodology, and reflexivity, and demonstrates how they can be operationalized in epidemiological practice. Feminist epidemiology emphasizes problem definition grounded in marginalized experiences, co-production of knowledge, intersectional analysis of sex, gender, and other social determinants, and critical attention to power relations. It advocates for the integration of qualitative, quantitative, and mixed methods, including non-traditional data sources and inclusive language, while maintaining scientific rigor through reflexivity and contextual sensitivity.

**Conclusion:**

Incorporating feminist principles transforms epidemiology and Public Health into a socially accountable, reflexive, and justice-oriented discipline. Health is both biological and political, and evidence production must account for structural inequalities, marginalized voices, and experiential knowledge. Feminist epidemiology does not sacrifice methodological rigor. It expands its scope to include ethical, social, and participatory dimensions, promoting more inclusive, accurate, and equitable science.

## 1 Introduction

Epidemiology, as the study of the distribution and determinants of health in populations (1), has profoundly shaped public health knowledge and policy (2). While early epidemiology examined social determinants and collective conditions, since the 1990s its focus has narrowed to individualized, micro-level risk factors, such as behavior, biology, and lifestyle (1, 2). This shift toward predictive, molecular, and risk-based models has often obscured structural determinants and reinforced a biomedical paradigm (2, 3).

Critical and social epidemiologists, most notably Nancy Krieger, have reconnected the field to its social roots. Krieger's theory of embodiment demonstrates how underlying social structures, hierarchies, and power dynamics such as racism, classism, and sexism literally become inscribed in bodies, emphasizing that health is biologically expressed but socially produced (3–5). This work laid the foundation for social and critical epidemiology, which examines how political, economic, and cultural systems shape health outcomes and inequalities (2, 6–8).

Feminist scholars including Marcia Inhorn (9, 10), Lisa Whittle (11), and Pat Kaufert (12) have extended this critique, exposing epidemiology's inherent androcentric assumptions and its failure to engage with women's lived realities. Kaufert's provocative question, “*Experts speak and women listen. What would happen if the pathway of communication was reversed?”* (12), captures feminism's epistemological challenge: to reorient health research toward those most affected by inequality.

Central to feminist critique is the analysis of patriarchy as a system embedded in institutions, language, and culture that privileges men, particularly those aligned with hegemonic masculinity (13), while marginalizing women, gender-diverse people, and non-hegemonic men (14–16). In health contexts, patriarchy manifests through medicalization (17, 18), paternalism, and androcentrism, which have long undermined women's bodily autonomy and positioned male bodies as the biomedical norm (19–21). The professionalization of medicine and technological expansion further entrenched physicians' authority, reinforcing reductionist conceptions of women's health as primarily reproductive and pathological (22–24).

Feminist scholarship has countered these dynamics by advancing bodily autonomy, health literacy, and participatory care. It has illuminated how shame and exclusion are socially produced, for example, through menstrual stigma, beauty norms, or medical neglect (25, 26), and reframed public health around embodiment, experience, and equity. Core concerns include reproductive rights, sexual autonomy, and the reduction of gender-based violence in healthcare (19, 21), such as obstetric violence (27–29).

Despite decades of feminist critique, explicitly feminist epidemiology remains rare. Quantitative health research continues to treat feminist insights as peripheral, rarely integrating reflexivity, intersectionality, or power analysis into study design, with some exceptions (20, 22, 30, 31). This commentary responds to early feminist epidemiologists' calls to bridge the epistemological divide between positivist health sciences and feminist scholarship. It argues for the systematic integration of feminist principles into epidemiological practice toward a more inclusive and socially accountable science.

### 1.1 Aim

Given the fragmentary and sporadic use of feminist analysis in quantitative health research, the central aim of this work is to make feminist principles employable within epidemiologic research. Integrating feminist principles with epidemiology is challenging because traditional epidemiological methods prioritize quantitative measurement and population-level inference, which can obscure the complex, intersectional, and structural dimensions of gendered power relations emphasized in feminist theory. Reconciling these approaches necessitates methodological innovation that accounts for social context, inequities, and the multidimensional nature of health determinants. This commentary selectively integrates and reinterprets principles from across feminist scholarship to render them applicable within epidemiological contexts. It seeks to stimulate further inquiry into how statistical methods and quantitative approaches might be reconceptualized through feminist perspectives, encouraging the development of a more reflexive and inclusive epidemiology.

## 2 Toward feminist principles for epidemiology and public health

Feminist principles are interconnected, processual, and circular, making their separation into discrete components necessarily artificial (14, 32–34). Feminist methodology, moreover, resists fixed or universally reproducible frameworks, remaining contextual, adaptable, and continually evolving. For clarity, they are discussed here in sequence - epistemology, methodology, theory, methods, and reflexivity - while acknowledging their interdependence.

### 2.1 Feminist epistemology: disrupting dominance in knowledge creation

Building on Foucault's idea that knowledge and power are intertwined (35), feminist scholars challenge the positivist notion of value-free science, arguing that all knowledge is perspective-bound and socially situated (33). Historically, knowledge production has been dominated by privileged White men from the Global North, whose worldviews have defined scientific inquiry, including assumptions about gender, health, and human nature (33, 36). This Eurocentric and colonial legacy established hierarchies privileging men, white persons, and heterosexuals while devaluing women's and marginalized knowledge systems (32). Feminist epistemology seeks to expose and destabilize these hidden processes by questioning – and eventually reframing – how, what, and who produces knowledge (37). Simply adding women to existing frameworks is insufficient; research must begin from women's and marginalized individuals lived experiences and knowledge (12, 20, 33). Feminist epistemology challenges dominant modes of knowledge production by amplifying marginalized voices and recognizing experiential knowledge as a legitimate and essential source of insight, thereby fostering more inclusive and reflexive forms of scientific inquiry (38, 39).

### 2.2 Feminist methodologies

#### 2.2.1 Feminist-informed problem definition

Feminist problem definitions center on health issues shaped by gendered oppression, exclusion, or neglect, reflecting topics often silenced by stigma or deemed socially irrelevant (9, 22). Conditions like endometriosis exemplify such neglect, remaining under-researched despite high prevalence (40). Feminist epidemiology therefore prioritizes overlooked populations and health issues that arise from structural inequities—such as gender-based violence, reproductive health, and the health impacts of unpaid care work—while challenging androcentric assumptions in study design and data interpretation to advance more just and context-sensitive understandings of health (10).

#### 2.2.2 Co-production of knowledge

Feminist research emphasizes collaboration with participants rather than research on them (36, 39). Researchers act as facilitators, valuing participants as knowledge producers (41). In epidemiology, inclusion can occur via expert panels, Delphi techniques, or “experts by experience,” meaning individuals whose lived experience of a health condition or social context provides valuable, context-specific insight that complements traditional scientific expertise (22, 37). For example, in research on autistic reproductive health, an insider-only Community-Partnered Participatory Research Council co-governed study design, recruitment, and communication, ensuring that autistic perspectives directly shaped the research process (23). When direct participation is limited, researchers can draw from qualitative evidence, community narratives, or social media to reveal research gaps and refine analyses (30). Co-production of knowledge ensures that marginalized voices shape research questions, interpretation, and dissemination.

### 2.3 The role of theory

Epidemiology often underuses theory, limiting reflexivity (24, 42). Feminist approaches advocate for theoretical engagement from the outset to clarify assumptions about gender, race, and inequality (43, 44). Drawing on sociology, medical anthropology, psychology, or educational sciences grounds interpretation in social context, making theory essential for robust and reflexive epidemiological research.

#### 2.3.1 Embracing complexity: sex, gender, and intersectionality

Sex and gender shape health outcomes, behaviors, and treatment responses (44, 45). Feminists critique conflation of sex and gender, which fosters essentialism (the assumption that men and women have inherent, fixed traits) (44, 46). Effective analysis distinguishes biological sex from sociocultural gender and includes sexual and gender diverse populations (47). Theories such as *doing gender* (the idea that gender is actively performed in everyday interactions) (48), relational gender theory (emphasizing how gender is shaped in relationships) (49), and hegemonic masculinity (dominant cultural ideals of masculinity that structure power) (13) reveal how gender operates across personal, institutional, and structural levels. Intersectionality further highlights how race, class, and sexuality intersect to produce unique health outcomes (31, 50–53). It is essential that feminist epidemiology integrate intersectional, theory-driven understandings of sex and gender in study design and interpretation (39). Feminist theory emphasizes that patriarchal power dynamics operate at macro, meso, and micro levels (16). Yet, structural analyses of gender in quantitative public health remain limited (54, 55). Feminist epidemiology critically examines how power relations and gender norms shape health inequalities across these dimensions (56).

### 2.4 A feminist approach to methods

#### 2.4.1 A call for feminist researchers to engage with quantitative methods

Feminist research has traditionally prioritized lived experiences and amplified marginalized voices, aligning naturally with qualitative methods (57, 58). However, feminist scholars increasingly advocate for the strategic use of quantitative and mixed methods to advance feminist inquiry (20, 39, 50, 57, 59). Quantitative methods can be enhanced by reflexively constructing variables and survey instruments that reflect lived experiences, for example, including multiple sex and gender options and open-text fields for self-identification validated with community input and auditing existing datasets for exclusionary sampling or culturally biased measures (39, 57). Theory-informed modeling, such as integrating intersectional frameworks into regression analyses, further ensures that analyses capture how gender, race, class, and other axes of inequality interact to shape health outcomes (20, 57). Feminist epidemiology maintains rigor by combining traditional epidemiologic standards with strategies from qualitative research, including reflexive documentation of research decisions, triangulation of data sources, and purposive inclusion of diverse perspectives. Collaborative interpretation with participants or community panels further reduces bias and ensures that findings are credible, contextually grounded, and responsive to the experiences of marginalized groups.

#### 2.4.2 Mixed methods and triangulation

Epidemiology often relies on secondary data and standardized measures, which can risk detachment from lived realities (12). Feminist approaches address this gap through methodological triangulation, integrating qualitative insights with quantitative datasets, including unconventional sources such as social media narratives, patient blogs, or community-generated data, to enrich interpretation, capture context, and highlight diversity (9, 30, 60–63). Sequential explanatory designs and co-interpretation with community or patient panels allow researchers to explain unexpected statistical patterns and ensure that marginalized perspectives actively shape interpretation and theoretical insights (36).

#### 2.4.3 Language as methodological practice

Language itself is a methodological tool. Feminist research emphasizes inclusive and participant-informed terminology, replacing terms such as “victim” with “survivor” or “delivered” with “gave birth” to reflect agency and dignity (39, 64, 65). Context-sensitive questionnaires, culturally and linguistically appropriate instruments, and iterative feedback from marginalized groups further enhance methods by increasing validity, reducing harm, and ensuring that findings remain grounded in participants' lived experiences (66, 67).

### 2.5 Feminist positionality: practicing reflexivity throughout the research process

Reflexivity, understood as the critical self-examination of the researcher's role, assumptions, and influence throughout the research process, is central to feminist research (36). It extends to awareness of broader social contexts, the researcher's own positionality, and relationships (37).

Feminist positionality recognizes how one's identity, including gender, race, sexual orientation, class, and ability, influences research. Continuous reflexivity helps researchers understand power dynamics, embodied consequences, and biases, fostering recognition of the researcher as a contextually situated individual (33, 37).

## 3 Discussion

Feminist scholarship offers powerful tools for reimagining epidemiology and Public Health as engaged, reflexive, and justice-oriented. By interrogating who produces knowledge, whose experiences are counted, and how structural power shapes health, feminist principles expose the limits of value-neutral, positivist inquiry. Integrating these principles does not compromise rigor. It redefines it, linking methodological precision with ethical and social awareness.

Feminist epidemiology can be advanced through collaboration between epidemiologists and feminist scholars or by adopting a feminist lens directly in research practice. This involves co-developing research questions with marginalized communities, constructing variables that capture gender, intersectionality, and social context, triangulating quantitative data with qualitative insights, and interpreting findings reflexively to highlight structural inequities. Such collaboration strengthens the rigor, relevance, and ethical grounding of epidemiological research, ensuring that it is socially accountable and responsive to the lived realities of diverse populations.

Feminist epidemiology recognizes health as both biological, social, and political, acknowledging that data and bodies are situated, and that inclusion begins not with representation alone but with the redefinition of research questions, measures, and interpretations. Making feminist principles employable in epidemiology and Public Health transforms evidence production from a tendency of detached observation to accountable knowledge creation. This approach invites a new generation of epidemiologists to build a science that is empirically robust, reflexive, and deeply responsive to the inequities it seeks to address.

## Data Availability

The original contributions presented in the study are included in the article/supplementary material, further inquiries can be directed to the corresponding author.
